# Electrical Resistance Activation of Embedded Fe-SMA Rebars in Pre-Cracked UHPFRC Beams: Internal Temperature Evolution and Calibrated Electro-Thermal Simulation

**DOI:** 10.3390/ma19102163

**Published:** 2026-05-21

**Authors:** Alireza Tabrizikahou, Jan Białasik, Karol Nowak, Krzysztof Lehmann, Grzegorz Trzmiel, Arkadiusz Dobrzycki

**Affiliations:** 1 Institute of Building Engineering, Poznan University of Technology, Piotrowo 5, 60-965 Poznan, Poland; jan.bialasik@put.poznan.pl (J.B.); krzysztof.lehmann@student.put.poznan.pl (K.L.); 2 Institute of Electric Power Engineering, Poznan University of Technology, 60-965 Poznan, Poland; karol.nowak@put.poznan.pl; 3 Institute of Electrical Engineering and Electronics, Faculty of Control, Robotics and Electrical Engineering, Poznan University of Technology, Piotrowo 3A, 60-965 Poznan, Poland; grzegorz.trzmiel@put.poznan.pl (G.T.); arkadiusz.dobrzycki@put.poznan.pl (A.D.)

**Keywords:** Fe-SMA, electrical resistance activation, fiber-reinforced high-performance concrete, activation temperature, electro-thermal simulation

## Abstract

Iron-based shape memory alloy (Fe–SMA) rebars can generate internal prestress in cement-based members after restrained thermal activation; however, the temperature actually reached by embedded rebars in cracked UHPFRC is difficult to infer from exposed bar segments. This study investigates electrical resistance activation of 4% prestrained Fe–SMA rebars embedded in pre-cracked UHPFRC beams and clarifies the activation-control problem by combining thermocouple measurements with a calibrated two-dimensional electro-thermal simulator. Twelve beams (150 × 150 × 600 mm) containing either Dramix 3D or Dramix 4D hooked steel fibers were first loaded in three-point bending to a mid-span displacement of 4 mm. The 4D series reached a 9.47% higher average pre-cracking load, confirming that fiber geometry modified the cracked state before heating. During activation, the exposed rebar segment reached 200 °C after approximately 77 s, whereas the embedded working segment reached the same target only after approximately 213 s; at that moment, the exposed segment was already close to 350 °C. The calibrated simulator reproduced the target activation time with an error of approximately 3 s and visualized the localized heat transfer from Fe–SMA to UHPFRC. The results demonstrate that activation control based only on exposed-bar temperature may cause under-activation of the embedded reinforcement, and that direct internal temperature monitoring is required for reliable Fe–SMA activation in cracked UHPFRC members.

## 1. Introduction

The ageing of reinforced-concrete infrastructure has increased the need for strengthening systems that can extend service life while limiting demolition, additional dead load, construction interruption, and environmental impact. Conventional strengthening strategies, including externally bonded steel plates, fiber-reinforced polymer laminates, reinforced-concrete jacketing, and added passive reinforcement, can improve load-bearing capacity, but their contribution is mainly passive. They generally do not introduce an active recovery force after damage, and their performance may be affected by corrosion, adhesive degradation, fire sensitivity, limited ductility, or premature debonding. For this reason, smart materials have received increasing attention in civil engineering, particularly when they can provide active prestressing, self-centering, crack-width control, vibration mitigation, or post-damage functionality rather than only increasing sectional resistance [1,2,3].

Shape memory alloys (SMAs) are among the most promising smart metallic materials for this purpose. Their distinctive behavior originates from a reversible martensitic phase transformation, which enables two functional mechanisms: the shape memory effect (SME) and superelasticity. In the SME, a previously deformed SMA element tends to recover its initial shape when heated above the transformation-temperature range. If this recovery is restrained, the imposed constraint is converted into recovery stress. In superelasticity, large recoverable strains can be obtained through stress-induced transformation and unloading, usually without the need for external heating. These mechanisms explain why SMAs have been investigated for active confinement, seismic self-centering, vibration control, prestressed strengthening, crack closing, and smart cementitious composites [1,2,4,5,6]. In concrete structures, the SME is especially relevant because it allows a prestrained metallic element to be installed in a member and later activated thermally to generate internal prestress without hydraulic jacks or conventional prestressing tendons.

Historically, many civil-engineering applications of SMAs were based on NiTi alloys because of their large recoverable strain, stable superelastic response, and well-established thermomechanical behavior. However, the high material cost and practical limitations associated with producing large structural NiTi components have restricted their widespread use in ordinary civil infrastructure. Iron-based shape memory alloys (Fe–SMAs), particularly Fe–Mn–Si-based systems modified with Cr, Ni, C, Nb, or V, offer a more realistic route for large-scale construction because they combine recovery-stress capability with comparatively low cost, weldability/formability, and compatibility with bar [7], strip, plate, and fiber-like reinforcing forms [8,9,10]. In these alloys, prestraining produces stress-induced martensite, and subsequent heating promotes reverse transformation and recovery-stress development when the element is restrained. Although the recoverable strain of Fe–SMA is generally lower than that of NiTi, the recovery stress that can be mobilized under restraint is sufficient for prestressed strengthening and crack-control applications in concrete and steel structures [11,12,13,14,15].

The transition of Fe–SMA from material-level characterization to structural application has been substantial during the last decade. Fe–SMA strips and plates have been used for prestressed strengthening of reinforced-concrete and steel members; near-surface-mounted and shotcrete-embedded Fe–SMA bars have been proposed for flexural strengthening; ribbed Fe–SMA bars have been characterized for concrete prestressing; and practical bridge-strengthening applications have demonstrated that the technology is no longer limited to small laboratory specimens [16,17,18,19,20,21,22,23]. More recent studies have further extended the field to Fe–SMA rebars or wires in new concrete members, thin-walled UHPC components, numerical strengthening strategies, and multiphysical activation models [24,25,26,27,28,29,30]. These studies show that Fe–SMA can enhance stiffness, crack resistance, residual capacity, and serviceability by transforming thermal activation into mechanical prestress.

In parallel with bar- and strip-based strengthening systems, Fe–SMA has also started to be explored in the form of short fibers and distributed active reinforcement. This concept is attractive because randomly dispersed Fe–SMA fibers can, in principle, generate localized recovery forces throughout a cementitious matrix and thereby contribute to crack bridging, crack-width restraint, post-damage strengthening, and self-healing-oriented behavior. Recent work has reviewed the experimental and computational state of SMA fiber-reinforced concrete and highlighted the need for reliable micromechanical and multiscale models [31]. Experimental investigations on Fe–SMA fibers have shown that fiber geometry, hook configuration, concrete matrix quality, thermal treatment, and loading rate govern pull-out resistance and load transfer [32,33]. Other recent studies demonstrated that thermally activated short Fe–SMA fibers can prestress concrete, improve flexural response, and partly restrain crack widening after damage [34,35]. These findings are important for the present work because they confirm that the interaction between Fe–SMA, the cementitious matrix, thermal activation, and cracking cannot be treated as a purely material-level issue; it is a coupled thermomechanical problem in which geometry, restraint, bond, and heat transfer all influence the final engineering effect.

Despite this progress, the activation stage remains one of the least standardized and most critical parts of Fe–SMA implementation. In externally accessible systems, the active element can often be heated and monitored directly, and surface temperature can be used as a reasonable control variable. In embedded systems, however, the temperature actually reached by the working part of the Fe–SMA is governed not only by electrical current and voltage, but also by concrete cover, thermal conductivity and heat capacity of the surrounding matrix, moisture condition, longitudinal heat flow, terminal/contact losses, boundary convection, and the crack state of the host member. Consequently, the temperature of an exposed rebar segment may not represent the temperature of the embedded segment where recovery stress is expected to develop. This problem is particularly relevant for electrical resistance activation, where Joule heating is generated in the metallic element but rapidly redistributed into the surrounding concrete [27,30]. If activation is controlled only by the exposed part of the rebar, the embedded portion may remain under-activated, whereas excessive heating of the accessible ends may create local overheating, oxidation, bond degradation, or unnecessary energy consumption.

The host cementitious material adds another level of complexity. Ultra-high-performance fiber-reinforced concrete (UHPFRC) is highly suitable for advanced repair and strengthening systems because of its dense matrix, high compressive and tensile performance, low permeability, and strong post-cracking capacity when steel fibers are used [36,37,38,39]. However, the same dense matrix and fiber-reinforced microstructure that improve mechanical and durability performance may also affect heat diffusion, moisture redistribution, thermal gradients, and crack-controlled transport during activation. Furthermore, the mechanical condition before activation is not independent of the UHPFRC fiber system. Hooked steel fibers influence crack bridging, residual flexural capacity, crack spacing, and local damage development [40,41]. Therefore, different steel-fiber geometries may create different pre-cracked states before Fe–SMA activation, even when the Fe–SMA rebars and electrical input remain nominally identical.

From this perspective, the specific scientific gap addressed in this study is not simply whether Fe–SMA can be activated, but whether reliable activation control can be achieved for prestrained Fe–SMA rebars embedded in pre-cracked UHPFRC beams. Existing literature has separately addressed Fe–SMA material behavior, prestressed strengthening with Fe–SMA bars or strips, activation guidelines for embedded Fe–SMA, UHPFRC mechanical performance, and Fe–SMA fiber/matrix interaction. However, limited information is available on the combined case in which prestrained Fe–SMA rebars are electrically activated inside a pre-cracked UHPFRC member, while the exposed and embedded rebar temperatures are monitored simultaneously and the surrounding UHPFRC contains different hooked steel-fiber geometries. The unresolved question is therefore whether the exposed rebar temperature can be safely used as an indicator of the activation state inside the concrete, or whether the embedded region must be monitored or predicted directly.

Accordingly, this study investigates the electrical resistance activation of 4% prestrained Fe–SMA rebars embedded in UHPFRC beams after controlled pre-cracking. Two UHPFRC series reinforced with Dramix 3D and Dramix 4D hooked steel fibers were considered to generate two different pre-activation cracked states. The target activation temperature of 200 °C was adopted based on the supplier documentation and the Fe–SMA literature as a practical temperature level for mobilizing recovery stress in the prestrained rebars [21,24,26]. The work is guided by three hypotheses: (i) the embedded rebar reaches the target activation temperature significantly later than the exposed rebar segment because of heat transfer into the surrounding UHPFRC; (ii) the 3D and 4D steel-fiber geometries mainly affect the pre-cracking response and the initial damage state, while their influence on the average activation temperature history is secondary relative to the effect of concrete cover and thermal inertia; and (iii) a calibrated two-dimensional electro-thermal model can reproduce the main activation milestone and help interpret the spatial heat field, but its predictive use for other geometries requires further validation.

The novelty of the study is therefore activation-control-related and methodological. First, the experiments directly compare exposed and embedded Fe–SMA rebar temperatures in pre-cracked UHPFRC beams, rather than relying on exposed-bar measurements or uncracked specimens. Second, the study evaluates activation after mechanically induced cracking in UHPFRC matrices containing two hooked steel-fiber geometries, thereby linking the pre-activation damage state to the thermal activation problem. Third, the experimental observations are supported by a transparent Python-based (version 3.11) electro-thermal simulator that visualizes localized heat diffusion and quantifies the delay between external and internal temperature development. The main contribution is to demonstrate that, for embedded Fe–SMA rebars in UHPFRC, activation should be controlled by the internal thermal state of the working rebar segment, or by a validated electro-thermal prediction, rather than by the temperature of an exposed rebar end alone.

## 2. Experimental Program

### 2.1. Constituent Materials and UHPFRC Matrix

The experimental campaign was carried out on UHPFRC beams with nominal dimensions of 150×150×600mm. The concrete matrix was designed as a dense, fine-grained UHPFRC without coarse aggregate, which is a common strategy for obtaining a compact microstructure, improved fiber dispersion, and high post-cracking performance in cement-based composites of this class [36,37,39].

The mix composition is summarized in Table 1. Portland cement CEM II 42.5R (provided by Holcim Polska located in Małogoszcz, Poland) was used as the primary binder. Limestone powder was introduced as a micro-filler to improve particle packing, whereas amorphous silica (SikaFume^®^ HR/TU provided by Sika-Poland located in Warsaw, Poland) was used as a reactive supplementary constituent to densify the cementitious matrix through pozzolanic action and to enhance the mechanical performance of the hardened material [36,37]. The granular skeleton consisted of two quartz-sand fractions, namely fine sand (0–0.5 mm) and coarse sand (0.5–1.0 mm), selected to achieve a dense packing of the matrix. A polycarboxylate-based superplasticizer (Sika^®^ ViscoCrete^®^-85 RS provided by Sika-Poland located in Warsaw, Poland) was used to ensure adequate workability at a low water-to-binder ratio, while a shrinkage-reducing admixture (SikaControl^®^-600 SRA provided by Sika-Poland located in Warsaw, Poland) was added to limit early-age shrinkage-related cracking.

**Figure 1:**
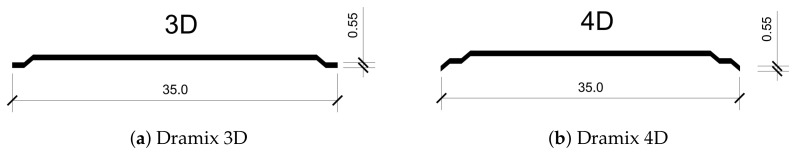
Geometry of the steel fibers (units in mm).

As dispersed reinforcement, hooked steel fibers were used at a dosage of 2.0% by volume of the mixture. Two commercially available geometries were considered: DRAMIX^®^ 3D 65/35BG and DRAMIX^®^ 4D 65/35BG (provided by NV Bekaert SA located in Zwevegem, Belgium). Both fiber types had a length of 35 mm, a nominal diameter of 0.55 mm, and an aspect ratio of 65 (see Figure 1). The main difference between them was the end-anchorage geometry, with the 4D fibers having more pronounced hooked ends. This distinction is important because hooked-end geometry strongly influences pull-out resistance, crack bridging, post-cracking load transfer, and residual flexural performance [39,40,41]. In the present study, the two fiber types were intentionally selected to assess whether differences in mechanical anchorage affect the pre-cracking response of the beams and, indirectly, the conditions under which the electro-thermal activation of the embedded Fe-SMA rebars is subsequently performed.

For the present beam series, visual inspection during casting did not reveal obvious fiber balls or severe segregation; however, no image-based fiber-orientation or dispersion index was determined. This should be considered when transferring the findings to larger members, because a UHPFRC mixture with 2.0 vol.% of 35 mm hooked fibers and a maximum aggregate size of 1 mm can be sensitive to mixing energy, casting direction and flow path. Future validation should therefore include fresh-flow testing, air-content measurement, mass-loss/moisture monitoring and section-level fiber-orientation assessment.

### 2.2. Fe–SMA Rebars and Beam Geometry

The active reinforcement consisted of commercially supplied ribbed Fe–SMA rebars (Fe–Mn–Si-based) with a nominal diameter of 10 mm (provided by re-fer AG located in Seewen, Switzerland). In the present work, the batch was specified through supplier technical documentation rather than through a new metallurgical characterization campaign. Therefore, the exact batch chemical composition, differential scanning calorimetry (DSC) response and production route were not independently measured. The available characteristics used for design and interpretation are summarized in Table 2.

Prior to casting, the rebars were prestrained to 4%, since prestraining is required for the subsequent generation of recovery stress during thermal activation [21,26]. Activation to 200 °C was adopted as the target because this value corresponded to the intended nominal prestressing force in the available rebar documentation and is consistent with previous Fe–SMA rebar studies in which heating to approximately 160–220 °C generated practically relevant recovery stress in prestrained Fe–SMA reinforcement [24,42]. It should be emphasized that recovery stress was not measured directly inside the concrete beams in this study; the present article verifies the electro-thermal activation condition, while the post-activation mechanical response requires a separate experimental program.

**Figure 2:**
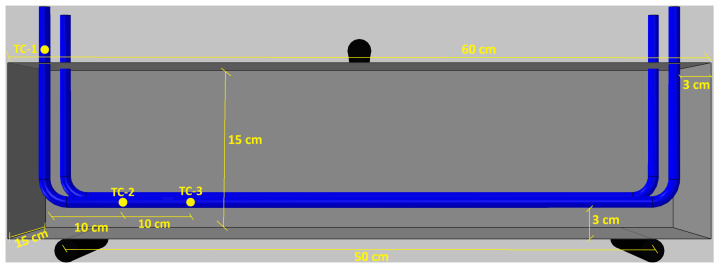
Beam geometry and Fe-SMA rebar arrangement.

The experimental program comprised two series, each containing six beams, resulting in a total of 12 specimens. The designation of the series is given in Table 3. The label identifies the type of steel fibers used in the UHPFRC matrix (Dramix 3D or Dramix 4D). Each beam contained two Fe–SMA rebars placed symmetrically in the tensile zone, with a concrete cover of 30 mm. The beam geometry and the arrangement of the Fe–SMA rebars are shown in Figure 2. The choice of an embedded-bar configuration, rather than an externally heated strengthening system, was intentional because the main objective of the study was to investigate the actual activation conditions of Fe–SMA rebars working inside a fiber-reinforced concrete member. This is particularly important in light of recent studies showing that the heating and prestressing behavior of Fe–SMA embedded in concrete differs significantly from that of bars exposed in air, owing to the effects of concrete cover, heat transfer, and boundary restraint [26,27,30].

### 2.3. Mixing, Casting, and Curing

The concrete constituents were batched and mixed under laboratory-controlled conditions following standard principles for the preparation of laboratory concrete specimens [43]. The dry powders and quartz-sand fractions were first homogenized, after which water containing the superplasticizer and shrinkage-reducing admixture was added progressively. The steel fibers were introduced gradually during the final mixing stage to reduce the risk of clumping. The fresh UHPFRC was cast into beam moulds with the Fe–SMA rebars fixed in their final positions before casting. Casting was performed along the beam length, which is relevant because the flow path may influence the orientation of the 35 mm hooked fibers.

After demoulding, all beams were cured in distilled water for 28 days and were subsequently stored for an additional 2 days under laboratory conditions before mechanical testing and electrical activation. The curing procedure was intended to ensure proper hydration of the dense cementitious matrix while keeping the beams in a moisture condition representative of laboratory testing. However, the exact internal moisture content before activation was not measured. For this reason, moisture-related observations during heating are interpreted qualitatively and are not used to quantify an independent hygro-thermal material parameter.

### 2.4. Pre-Cracking by Three-Point Bending

**Figure 3:**
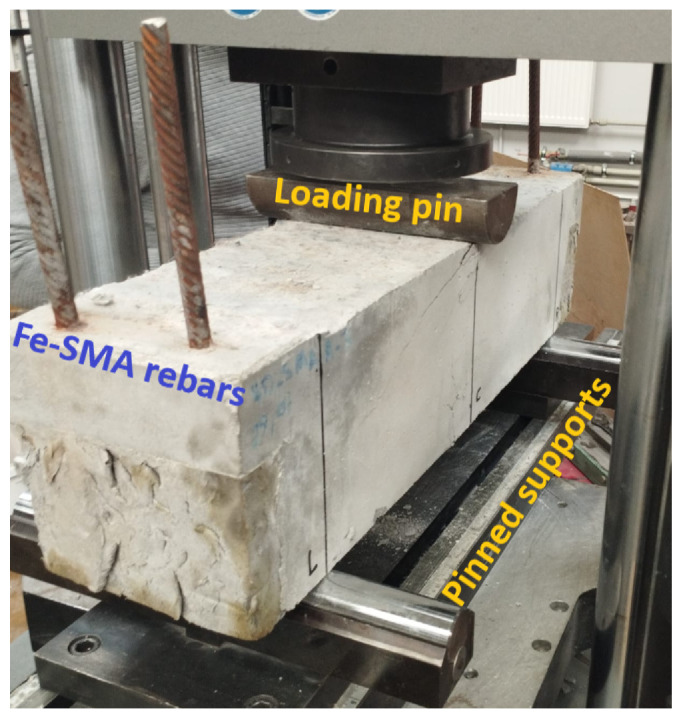
UHPFRC beam with Fe-SMA rebars during three-point bending test.

In the first experimental stage, each beam was subjected to three-point bending in order to introduce a controlled and relatively mild level of damage before activation of the Fe–SMA rebars. The tests were performed with a support span of 500 mm and a displacement-controlled loading rate of 2 mm/min, as shown in Figure 3.

The target mid-span displacement in this stage was set to 4 mm. This value was established on the basis of preliminary pilot tests performed on beams of identical geometry and matrix composition reinforced with 3D and 4D steel fibers but without Fe–SMA rebars. The purpose of this preliminary calibration was to determine a displacement level sufficient to generate measurable flexural cracking while avoiding severe degradation of the beam before activation. Once the prescribed displacement of 4 mm was reached, the test was stopped and the beam was unloaded. This procedure provided a reproducible pre-cracked state that served as the starting condition for the subsequent electrical resistance activation of the Fe–SMA rebars.

The adopted pre-cracking strategy was especially important for the present study because its purpose was to create a representative lightly damaged state prior to activation. In this study, pre-cracking was introduced to simulate a realistic service condition of a cracked UHPFRC member with embedded Fe–SMA rebars. The objective was not to assess the post-activation mechanical response of the beams, but to study the internal and external temperature evolution of the rebars during electrical resistance activation under this controlled cracked state.

During this stage, the applied force and mid-span displacement were recorded continuously. The resulting load–displacement curves are reported as average responses for the six beams in each fiber series. Crack width maps, residual deflection after unloading and quantitative crack-area measurements were not recorded in a form suitable for statistical post-processing. The pre-cracking data should therefore be interpreted as defining a controlled damage level, not as a full fracture-mechanics characterization of the beams.

### 2.5. Electrical Resistance Activation Setup

After the pre-cracking stage, all beams were subjected to electrical resistance heating of the embedded Fe–SMA rebars. Electrical resistance heating was selected because it enables localized thermal activation of the alloy without heating the entire concrete element, which is one of the main practical advantages of Fe–SMA activation in concrete members [26,27].

**Figure 4:**
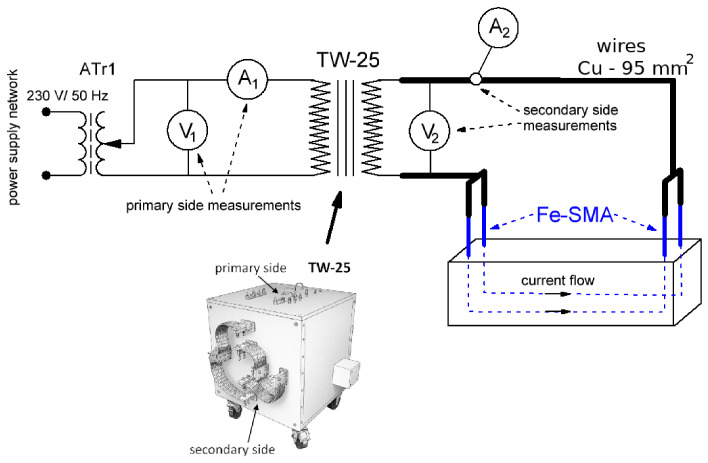
Schematic diagram of the electrical resistance activation setup for Fe-SMA elements: Tr1—autotransformer; TW-25—high-current transformer; V1—primary voltage measurement; A1—primary current measurement; V2—secondary voltage measurement; A2—secondary current measurement; T—temperature measurement system (Fluke 54 II B and PicoLog with K-type thermocouples). The secondary side was connected to the Fe-SMA element using Cu 95 mm^2^ cables.

A schematic view of the activation system is shown in Figure 4. The setup consisted of an autotransformer (Tr1), a high-current transformer TW-25, a primary-side measurement circuit, a secondary-side measurement circuit, and the heating circuit formed by the Fe–SMA rebars. The operating principle was based on regulating the voltage supplied to the primary winding of the high-current transformer TW-25. By adjusting the output of the autotransformer Tr1, the primary voltage of the TW-25 could be varied, which in turn changed the secondary voltage and the current flowing through the Fe–SMA rebars. Such a configuration is typical of high-current systems because the regulation is carried out on the lower-current primary side, which simplifies control and reduces losses in the regulation path [44].

**Figure 5:**
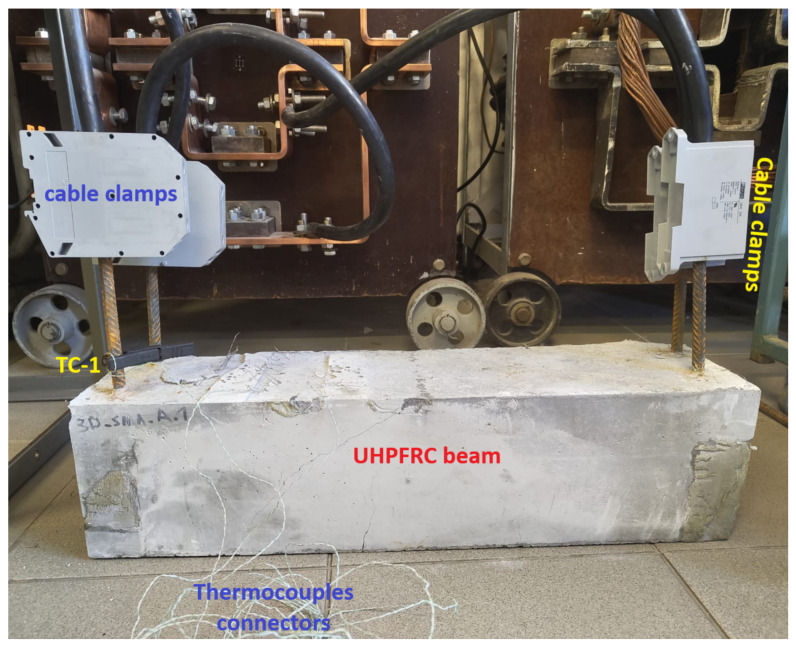
UHPFRC beam connected to the electrical resistance activation setup: visible Cu 95 mm^2^ cable clamps on the protruding ends of Fe-SMA rebars.

The TW-25 unit acted as a source of low voltage and high current, which is necessary for resistance heating of metallic elements with very low electrical resistance. The transformer was supplied at 230 V/50 Hz and had a rated power of 25 kVA. Owing to its sectional winding arrangement, it allowed different operating configurations depending on the required secondary current range. A view of a beam connected to the activation setup is shown in Figure 5.

On the primary side of the TW-25 transformer, the supply voltage and current were monitored continuously. The primary voltage was measured using instrument V1, while the primary current was measured using instrument A1; in both cases, OWON D35T multimeters (by Owon located in Zhangzhou, China) were used, operating respectively in voltmeter and ammeter mode. On the secondary side, the voltage applied to the heating circuit was measured with instrument V2 (UNI-T UT71E by Uni-Trend Technology located in Dongguan, China), whereas the current flowing through the Fe–SMA rebars was monitored with current clamp A2 (Sonel CMP-2000 by Sonel S.A. located in Świdnica, Poland). The secondary circuit was connected to the protruding ends of the Fe–SMA rebars using Cu 95 mm^2^ copper cables. The large cross-section of these conductors was intentionally selected to minimize voltage drops and parasitic Joule losses in the supply cables, thereby maximizing the fraction of electrical energy dissipated directly in the heated rebars.

From an electro-thermal point of view, the resistive activation of Fe-SMA rebars can be described by the same energy-balance framework that is classically used for current-carrying conductors and busbars. The heat source is Joule dissipation in the metallic element, which in the first approximation may be written as $P_{J}\left(t\right)=I^{2}\left(t\right)R\left(t\right)\approx U\left(t\right)I\left(t\right)$. The temperature rise results from the competition between the power generated in the rebar and the heat transferred to the surrounding medium. In lumped form, the process may be expressed as:

(1) $$C_{th}\frac{d\Delta \Theta }{dt}=P_{J}-G_{th}\Delta \Theta$$

where $\Delta \Theta =T-T_{\infty }$, $C_{th}$ is the effective thermal capacity of the system, and $G_{th}$ is the effective thermal conductance to the surroundings. For quasi-constant electrical loading, this yields the exponential heating law:

(2) $$\Delta \Theta \left(t\right)=\Delta \Theta_{ss}\left(1-e^{-\frac{t}{T_{th}}}\right)+\Delta \Theta \left(0\right)\cdot e^{-\frac{t}{T_{th}}}$$

where $\Delta \Theta_{ss}=\frac{P_{J}}{G_{th}}$ and $T_{th}=\frac{C_{th}}{G_{th}}$. Thus, in the present study, the heat-generation mechanism is the same as in classical conductor-heating problems, whereas the boundary conditions are markedly different: the Fe-SMA rebar is thermally coupled to a massive UHPFRC matrix, which acts both as an additional heat sink and as a source of thermal inertia, while the presence of moisture further modifies the transient response.

The activation tests were performed at an average secondary current of 420 A and an average secondary voltage of 2.6 V. For interpretation of the heating process, these values were treated as RMS quantities corresponding to an approximately resistive load of the Fe-SMA activation circuit. These values were sufficient to reach the target activation temperature of 200 °C in the rebars embedded in the concrete beam.

The average values recorded on the secondary side correspond to an effective circuit resistance of approximately $R_{\mathrm{eff}}=U/I=2.6/420\approx 6.2\;m\Omega$ and an average electrical input power of approximately P=UI≈1.09kW. For the heating duration required to reach the embedded activation target, this corresponds to an electrical energy input of about 230–235kJ. This is a circuit-level value and includes the combined contribution of rebars, cable connections and terminal contact effects; the individual contact resistance at the clamps was not measured separately. The use of RMS voltage and current was considered appropriate because the heated element behaved predominantly as a low-resistance metallic load under 50 Hz supply, but waveform distortion and phase shift were not recorded.

The external rebar segment reached temperatures close to 350 °C by the time the embedded segment attained the target value. This overheating of the exposed segment is not representative of the embedded working length, but it is relevant from a durability and operational-safety perspective. Prolonged or repeated overheating of exposed Fe–SMA may promote surface oxidation and could modify local terminal contact conditions. Therefore, the external segment temperature should be monitored as a safety variable, whereas the internal rebar temperature should be used as the activation-control variable.

### 2.6. Temperature Monitoring and Activation Criterion

**Figure 6:**
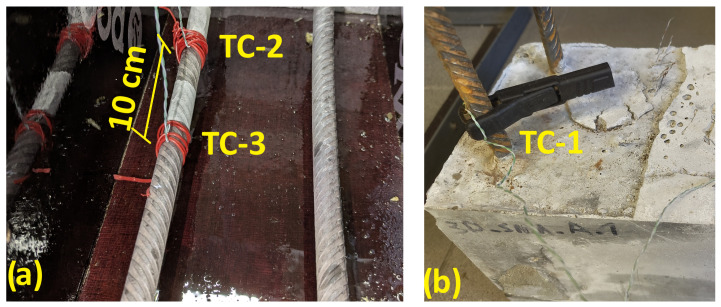
Thermocouple installation: (**a**) thermocouples TC-2 and TC-3 attached to Fe-SMA rebars inside the formwork before casting; (**b**) thermocouple TC-1 on the external segment of the Fe-SMA rebar during electrical resistance activation.

The temperature of the Fe–SMA rebars during activation was monitored using two complementary measurement paths. Preliminary temperature control was carried out using a Fluke 54 II B (by Fluke Corporation located in Everett, WA, USA) contact thermometer, whereas the main time-dependent temperature recording was performed using K-type thermocouples connected to a PicoLog data-acquisition system. Three thermocouples were used in total. Two thermocouples, denoted TC-2 and TC-3, were attached directly to the Fe–SMA rebars inside the beam before casting, at a mutual spacing of 10 cm. The third thermocouple, denoted TC-1, was mounted on the exposed segment of the rebar outside the concrete and served as a reference point. The thermocouple arrangement is shown in Figure 6.

The activation procedure was intentionally conservative. The heating process was terminated only when the internal thermocouple TC-2 reached 200 °C. This criterion was adopted because the temperature of the rebar embedded in concrete, rather than the temperature of the exposed rebar segment, governs the effectiveness of activation in the actual structural element. Simultaneous monitoring of both the internal and external rebar temperatures enabled not only safe control of the process, but also direct assessment of the difference in heating rate between the exposed and embedded zones. This point is methodologically important because recent studies have shown that the thermal response of embedded Fe–SMA is strongly influenced by concrete cover, thermal boundary conditions, and the coupled heat-transfer process in the surrounding cementitious matrix [26,27]. In the present case, this approach also reduced the risk of under-activating the working part of the Fe–SMA rebar inside the beam while unintentionally overheating its exposed ends. The adopted temperature-control strategy therefore allowed the activation protocol to be referenced to the actual thermal state of the Fe–SMA inside the concrete member, which was the central methodological requirement of the present study.

The thermocouples were fixed to the Fe–SMA rebars before casting to maintain direct thermal contact with the metallic reinforcement during activation. The recorded temperatures therefore represent local contact measurements at the thermocouple locations rather than a continuous temperature field along the whole rebar. Since only three thermocouples were used, the spatial resolution of the experimental monitoring was limited. Thermal lag of the thermocouple junctions, local debonding, and small differences in contact pressure could also contribute to measurement uncertainty. These limitations motivated the use of the numerical model as an interpretive tool for visualizing the thermal field between measurement points.

## 3. Experimental Results

### 3.1. Initial Flexural Response Prior to Fe–SMA Activation

**Figure 7:**
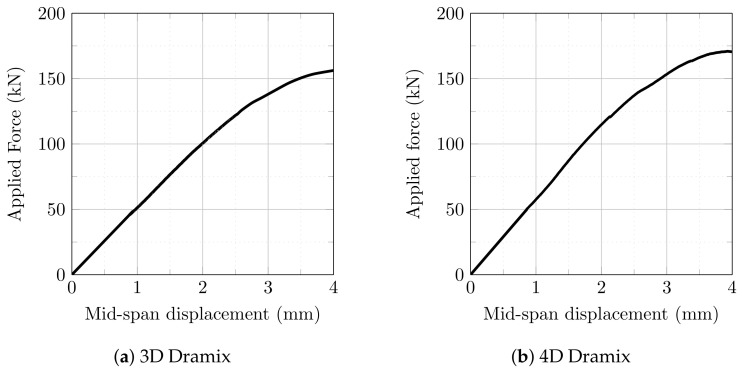
Average load–displacement curves during the pre-cracking stage for beams with 3D and 4D fibers.

Figure 7 presents the average load–displacement response obtained during the pre-cracking stage, based on six beams reinforced with Dramix 3D fibers and six beams reinforced with Dramix 4D fibers. The purpose of this stage was to document the initial cracked condition prior to activation, not to evaluate post-activation structural performance. Beams reinforced with Dramix 3D fibers reached an average flexural load of 156.12 kN, whereas beams reinforced with Dramix 4D fibers reached 170.91 kN, corresponding to an increase of 9.47% in favor of the 4D series. This difference is consistent with recent direct pull-out tests on hooked Fe–SMA fibers embedded in high-performance concrete, where 4D double-hook fibers developed approximately 50–70% higher peak pull-out forces than 3D single-hook fibers under ambient conditions [33]. This supports the interpretation that the more pronounced 4D end-hook geometry improves mechanical anchorage and crack-bridging efficiency. Nevertheless, the beam-scale difference observed here should still be interpreted cautiously, since fiber distribution, casting-induced orientation, and local matrix–fiber bond variability may also contribute to the measured pre-cracking response.

This difference can be plausibly attributed to the more developed end-anchorage geometry of the 4D fibers, which likely provided more efficient mechanical interlock with the matrix, improved crack-bridging action, and delayed crack opening under bending. Such an interpretation is consistent with previous studies showing that the end-hook geometry of steel fibers has a pronounced effect on flexural strength, toughness, and post-cracking behavior, with increasingly developed hook geometries generally leading to better structural performance [40,41]. This trend is also in agreement with broader review studies on UHPFRC/UHPC, which emphasize that fiber geometry, fiber–matrix interaction, and pull-out resistance are among the key parameters governing tensile and flexural response [37,39]. From the perspective of the present investigation, this result is important mainly because it defines the cracked state in which the activation process was performed. Thus, the comparison between 3D and 4D fibers serves primarily to document the structural condition prior to heating rather than to claim a post-activation mechanical benefit within the scope of the present paper.

Because replicate-level force–displacement data were not available in a form that allowed reliable reconstruction of error bands, Figure 7 is retained as an average-response comparison and no inferential statistical test is claimed. The observed 9.47% difference is therefore interpreted descriptively. Although the more pronounced hook geometry of the 4D fibers provides a plausible mechanical explanation, possible contributions from local fiber dispersion, casting direction, and fiber–matrix bond variability cannot be completely excluded.

### 3.2. Visual Observations During Electro-Thermal Activation

**Figure 8:**
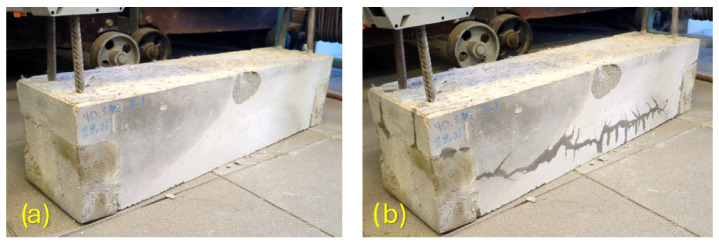
UHPFRC beam with Dramix fibers during electrical resistance activation: (**a**) before heating; (**b**) after heating—visible traces of moisture/steam escaping through micro-cracks.

During the activation stage, visible traces of moisture and vapor release through microcracks were observed on the beam surface, as illustrated in Figure 8. Although this observation is qualitative in nature, it provides useful insight into the coupled thermo-hygro-mechanical response of the pre-cracked UHPFRC beams during electrical resistance heating. The release of moisture through microcracks suggests that part of the supplied electrical energy was indirectly consumed by heating the surrounding concrete and by promoting evaporation of pore water in the vicinity of the heated rebars.

This observation is consistent with the current understanding of the thermal behavior of UHPC/UHPFRC. When such dense cementitious materials are exposed to elevated temperature, their response is strongly influenced by moisture transport, pore-pressure development, thermal gradients, and fiber–matrix interactions [45]. In the present case, the beams were not globally heated in a furnace; rather, the heat source was highly localized in the embedded Fe–SMA rebars. Even so, the observed moisture migration confirms that the thermal field generated by resistive activation extended beyond the rebars themselves and interacted with the surrounding cementitious matrix. This point is particularly relevant in pre-cracked members, where cracks and microcracks may act as preferred pathways for moisture migration and heat dissipation.

### 3.3. Temperature Evolution During Electrical Resistance Activation

**Figure 9:**
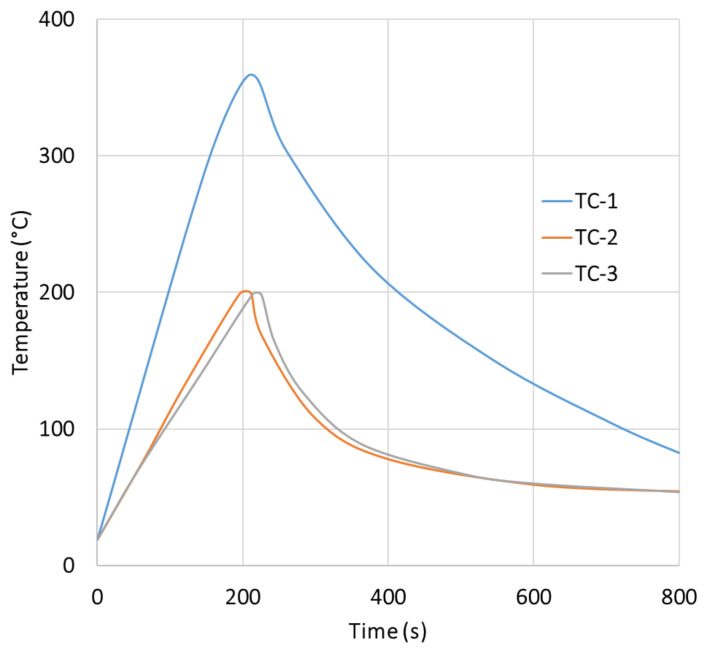
Average temperature–time histories for the external thermocouple (TC-1) and internal thermocouples (TC-2 and TC-3), considering all tested beams (the curves represent the mean trend obtained from the twelve specimens).

The most important result of the present study concerns the temperature evolution recorded during the electrical resistance activation of the Fe–SMA rebars embedded in the UHPFRC beams. No systematic difference was observed between the temperature–time responses of the 3D and 4D series during activation; therefore, Figure 9 presents the average temperature histories obtained considering all tested beams together. The external thermocouple TC-1, mounted on the exposed segment of the rebar, reached 200 °C after approximately 77 s. However, heating was not terminated at that point because the adopted control criterion required the internal thermocouple TC-2, embedded inside the beam, to reach the target activation temperature. As a result, the external segment continued to heat up and attained approximately 350 °C at the moment the power supply was switched off.

By contrast, thermocouple TC-2, located on the embedded rebar inside the beam, reached the target temperature of 200 °C only after approximately 213 s. Thermocouple TC-3, placed 10 cm away from TC-2 on the same embedded rebar, reached a temperature close to the target value within the same time interval. The difference between the external and internal heating times was therefore approximately 136 s, which clearly demonstrates that the thermal response of the exposed rebar segment cannot be considered representative of the thermal state of the working rebar portion embedded in the concrete.

Figure 9 should be interpreted as an averaged thermal response rather than as the result of a single representative specimen. The individual temperature records from the six 3D beams and the six 4D beams were compared before averaging in order to assess the repeatability of the heating process and the possible influence of steel-fiber geometry on the thermal response. No systematic separation was observed between the 3D and 4D series in terms of the overall heating trend, the attainment of the internal activation temperature, or the subsequent cooling branch. The scatter between individual records was mainly associated with local experimental factors, including thermocouple contact conditions, local crack distribution, moisture redistribution, and small differences in the electrical contact condition during activation, rather than with the steel-fiber geometry itself. Therefore, the robust conclusion drawn from Figure 9 is the existence of a substantial thermal lag between the externally exposed and internally embedded rebar segments, whereas minor differences between the 3D and 4D temperature histories should not be overinterpreted.

This finding is fully consistent with recent studies on embedded Fe–SMA activation, which have shown that the resistive heating process is strongly affected by concrete cover, bar diameter, current density, and geometric configuration [26,27]. In other words, the electrical parameters alone do not uniquely define the activation state of the alloy; rather, they must be interpreted together with the thermal boundary conditions imposed by the surrounding concrete. The present results confirm this point experimentally for pre-cracked UHPFRC beams and show that direct temperature control inside the element is essential if reliable activation is to be achieved.

The temperature histories shown in Figure 9 exhibit a quasi-exponential character, which is consistent with the classical first-order thermal response of a current-carrying element. In this interpretation, the difference between TC-1 and TC-2 does not arise from a different heat-generation mechanism, but from different effective values of $C_{th}$ and $G_{th}$. The external rebar segment behaves as a conductor cooled mainly by air, whereas the segment embedded in UHPFRC transfers a substantial part of the supplied energy into a much larger thermally active volume of the surrounding material. This increases the effective heating time constant and delays the attainment of the activation temperature. Such an interpretation is also consistent with the observed release of moisture through microcracks and explains why the temperature measured on the exposed segment cannot be regarded as representative of the working rebar length inside the member.

A further detail worth noting is the difference in the heating trajectories of TC-2 and TC-3. Although both thermocouples were mounted on the embedded rebars and were spaced only 10 cm apart, their temperature histories were not fully identical. In particular, the slower heating rate of TC-3 in the range of approximately 100–150 °C suggests local non-uniformity of the thermal environment inside the beam. A plausible explanation is the local variation in moisture content and microcrack distribution within the surrounding concrete, which may have altered the local heat-transfer conditions and temporarily absorbed part of the supplied energy through evaporation processes. Given the visible moisture release observed at the beam surface, this interpretation appears physically justified.

Table 4 summarizes the main activation indicators derived from the average temperature histories. The values are approximate because they are based on the averaged experimental curves and the adopted activation cutoff criterion. The difference between TC-2 and TC-3 was observed in the averaged temperature history, but the available records do not allow a specimen-by-specimen crack-pattern correlation. Therefore, the local non-uniformity is interpreted as an indication that small variations in moisture content, crack opening, thermocouple contact and local heat sink conditions can perturb the temperature history even over a distance of 10 cm. This experimental non-uniformity also explains why the symmetric two-dimensional model should be regarded as a cross-sectional average representation rather than as a point-by-point reproduction of every thermocouple response.

### 3.4. Engineering Implications of the Measured Activation Response

From an engineering standpoint, the results obtained in this study have direct implications for the design and control of activation procedures for embedded Fe–SMA rebars. First, they demonstrate that using the temperature of an exposed rebar segment as the sole control parameter would lead to a misleading assessment of the activation state. In the present tests, the exposed segment reached the target temperature more than twice as fast as the embedded segment and exceeded the nominal target temperature by approximately 150 °C at the moment when the internal rebar finally reached 200 °C. If the process had been stopped based only on the external thermocouple, the internal part of the Fe–SMA rebar would have remained under-heated and the intended activation level would not have been achieved.

Second, the results indicate that the thermal inertia of the concrete cover and the moisture present in the matrix substantially delay the temperature rise of the embedded Fe–SMA rebar. This observation reinforces the argument that activation procedures for concrete members should be based on direct measurement of the internal thermal state of the Fe–SMA, especially when the member has been pre-cracked or contains a dense steel-fiber-reinforced matrix. Recent activation-guideline studies have similarly emphasized that reliable resistive activation requires calibration for the specific bar geometry and concrete configuration under consideration [27].

Third, the present findings should be interpreted within the scope of this study. The tests were performed for one beam geometry, one concrete-cover thickness, one Fe–SMA bar diameter, and one target activation temperature. Therefore, the reported activation times and temperature differences should not be treated as universal values, but rather as experimentally verified values for the specific configuration studied herein. Nevertheless, the underlying trend is clear and robust: the internal thermal response of an embedded Fe–SMA rebar differs markedly from that of its exposed segment, and this difference must be taken into account in both experimental studies and practical applications.

Overall, the results show that the pre-cracking flexural response was influenced by steel-fiber geometry, whereas the subsequent electro-thermal activation response was governed primarily by the thermal interaction between the Fe–SMA rebars and the surrounding UHPFRC matrix. In this sense, the study provides an experimentally grounded basis for calibration of activation procedures and for future studies on the post-activation mechanical response of such members.

Although post-activation reloading was outside the experimental scope, the expected mechanical consequence of reaching the embedded rebar target temperature can be discussed qualitatively. If the prestrained Fe–SMA is sufficiently restrained by the surrounding concrete and anchorage, recovery stress should introduce an internal prestressing action. This action is expected to promote partial crack closure, reduce residual crack opening, increase decompression resistance, improve apparent flexural stiffness under service-level reloading, and enhance serviceability. These effects are inferred from the known mechanism of restrained Fe–SMA recovery and from previous structural studies, but they were not directly verified in the present beam tests.

The direct numerical values reported here should be considered valid primarily for beams with dimensions close to 150×150×600mm, a 30 mm cover, two 10 mm Fe–SMA rebars, similar UHPFRC thermal properties and a target temperature of 200 °C. Larger members, bridge-scale overlays, longer embedded lengths, thicker covers or larger rebar diameters will exhibit longer thermal time constants and different longitudinal heat losses. Field application should therefore not transfer the present activation time directly; instead, it should use internal temperature monitoring or a calibrated model updated for the actual cover, bar diameter, active length, ambient conditions and moisture state.

## 4. Electro-Thermal Simulation of Activation of Fe-SMA Rebars

To complement the experimental program, a custom Python application with an interactive Tkinter graphical user interface (GUI) was developed to simulate the electro-thermal response of the tested beam cross-section during Joule activation of the embedded Fe-SMA rebars. The model simulates transient heat generation in two circular Fe–SMA rebar domains and heat diffusion into the surrounding UHPFRC cross-section. It was calibrated by adjusting the effective source-gain factor so that the computed mean embedded-rebar temperature reached the experimental activation target at the measured activation time. The additional insight provided by the model is therefore spatial rather than purely curve-fitting: it visualizes how localized heat spreads from the rebars into the UHPFRC, identifies the high-gradient zones near the reinforcement, and enables controlled parameter studies on thermal properties and numerical settings. The purpose of the program was not to reproduce the full three-dimensional electro-thermo-mechanical behaviour of the beam, but to provide a transparent and user-controllable engineering tool capable of: (i) reproducing the experimentally observed heating rate of the Fe-SMA reinforcement, (ii) visualising the transient temperature field in the beam cross-section, and (iii) generating plots and animation frames suitable for interpretation and publication.

The developed tool models the beam cross-section as a two-dimensional domain composed of two materials only, namely UHPFRC and Fe-SMA. The analysed section had dimensions of 150×150mm and contained two Fe-SMA rebars with a nominal diameter of 10mm. Based on the experimental arrangement, the rebars were positioned symmetrically, with a clear side cover of 30mm and a clear bottom cover of 30mm. Consequently, the centres of the rebars were located at (x,y)=(35,35)mm and (115,35)mm, with a centre-to-centre spacing of 80mm. The electrically active length of each bar was assumed as 1.0m.

The spatial model presented below may be regarded as a distributed-parameter extension of the classical lumped conductor-heating model: Joule heat is generated in the Fe-SMA domain and is then dissipated by conduction into the UHPFRC matrix and by convection at the external boundaries of the cross-section.

### 4.1. Governing Formulation

Because the secondary circuit operated at 50 Hz, while the load at the heated Fe-SMA rebars was treated as approximately resistive, the electrical input to the model was defined from the measured RMS values. Accordingly, the total electrical power supplied to the system was taken as:

(3) $$P=U_{rms}\cdot I_{rms}$$

where $U_{rms}$ is the measured secondary RMS voltage and $I_{rms}$ is the measured secondary RMS current.

In the present implementation, the total power is divided equally between the two rebars, which is consistent with the symmetric parallel connection used in the experiments. The nominal volumetric heat source in one rebar is then written as

(4) $$q_{\mathrm{nom}}^{\prime \prime \prime }=\frac{P/2}{A_{s}L_{\mathrm{act}}},$$

where $A_{s}=\pi d^{2}/4$ is the rebar cross-sectional area, *d* is the rebar diameter, and $L_{\mathrm{act}}$ is the active heated length.

The transient temperature field is governed by the heat-conduction equation with internal heat generation:

(5) $$\rho c_{p}\frac{\partial T}{\partial t}=\nabla \cdot \left(k\nabla T\right)+q^{\prime \prime \prime },$$

where ρ is density, $c_{p}$ is specific heat capacity, *k* is thermal conductivity, *T* is temperature, and $q^{\prime \prime \prime }$ is the volumetric Joule-heating source term applied only inside the Fe-SMA domains. This form is consistent with the electro-thermal coupling used in the literature for Joule-heated Fe-SMA systems [30]. Heat exchange with the surroundings is represented by a convective boundary condition on the external edges of the beam section:

(6) $$-k\nabla T\cdot n=h\left(T-T_{\infty }\right),$$

where *h* is the convection coefficient and $T_{\infty }$ is the ambient temperature.

**Figure 10:**
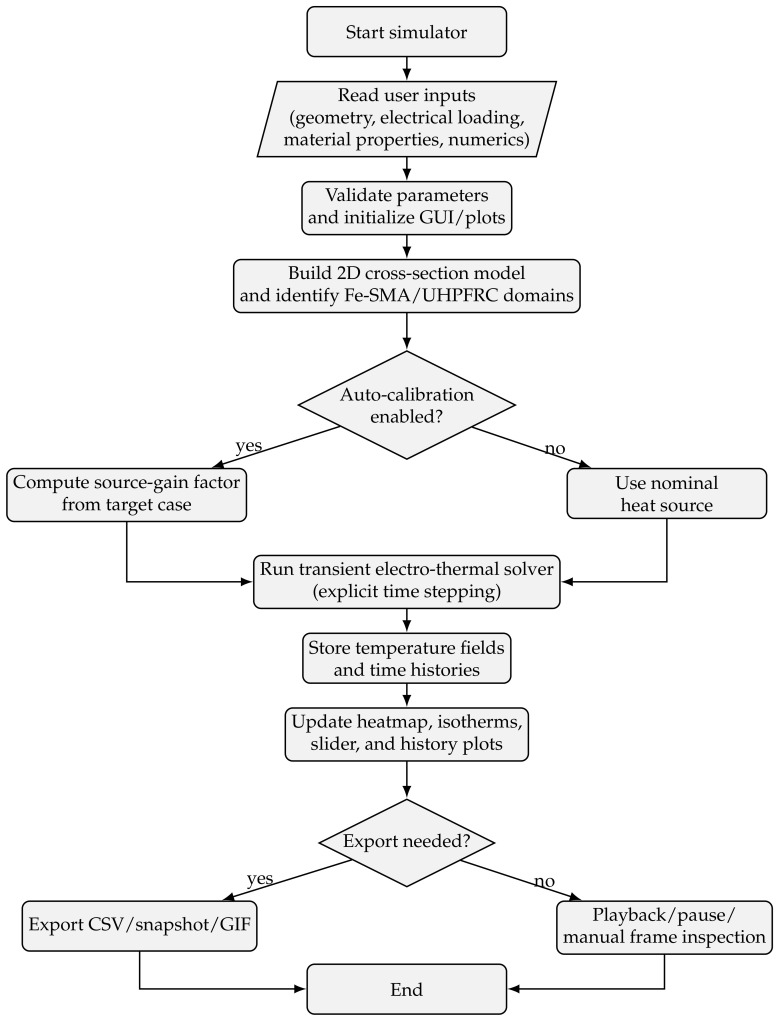
Schematic workflow of the custom Python electro-thermal simulator used for the activation analysis of embedded Fe-SMA rebars in UHPFRC beams.

To clarify the internal logic of the developed simulator, the workflow of the custom Python tool is summarized schematically in Figure 10. The flowchart shows the sequence from user-defined input and parameter validation, through model generation and optional source calibration, to transient electro-thermal solution, visualization of the temperature field and histories, and export of the generated outputs.

### 4.2. Numerical Implementation

The code solves the problem on a structured two-dimensional grid using an explicit transient finite-difference/finite-volume type scheme. In the reference simulations presented here, a mesh of 121×121 nodes was used, which provided a sufficiently smooth temperature field while maintaining short computation time on a standard personal computer. Spatially varying conductivity between UHPFRC and Fe-SMA was handled through harmonic averaging at the cell interfaces, which improves numerical stability at material boundaries. In the current implementation, the default time increment was Δt=0.01s, and the program automatically checks whether the selected time step satisfies the stability limit of the explicit scheme.

It should be emphasised that this model is a calibrated two-dimensional cross-sectional model. Therefore, several physical effects are not described explicitly, such as the longitudinal temperature gradient along the bar, heat losses through the protruding bar ends outside the concrete, local contact effects at the electrical terminals, and possible temperature dependence of electrical resistivity. To compensate for these simplifications and to reproduce the measured heating rate, the program introduces an effective source-gain factor *g*, obtained automatically from the reference experimental case:

(7) $$g=\frac{T_{\mathrm{target}}-T_{\infty }}{T_{\mathrm{rebar}}^{\left(g=1\right)}\left(t_{\mathrm{target}}\right)-T_{\infty }},$$

where $T_{\mathrm{target}}$ is the experimentally observed rebar temperature at time $t_{\mathrm{target}}$, and $T_{\mathrm{rebar}}^{\left(g=1\right)}\left(t_{\mathrm{target}}\right)$ is the numerically predicted mean rebar temperature obtained from a unit-gain simulation. The effective heat source is then

(8) $$q_{\mathrm{eff}}^{\prime \prime \prime }=g\;q_{\mathrm{nom}}^{\prime \prime \prime }.$$

In this way, the code remains simple and transparent, while still reproducing the experimentally relevant temperature level.

For transparency, the source-gain factor should not be interpreted as a physical heat-loss coefficient. It compensates collectively for effects that are outside the present two-dimensional formulation, including longitudinal temperature gradients, electrical terminal losses, nonuniform current paths, temperature-dependent resistivity, heat storage in the protruding rebar parts, and moisture-related processes in the surrounding UHPFRC. In the calibrated reference case, the simulation reached the target rebar temperature at approximately 210.6s, compared with the experimental internal activation time of approximately 213s. The corresponding activation-time difference is about 2.4–3.0s, or approximately 1–1.5% of the measured activation time. This metric verifies the calibration target only; it should not be confused with independent predictive validation.

A first-order sensitivity check was also performed by changing selected thermal parameters by ±20% while keeping the calibrated source factor unchanged. Increasing the UHPFRC thermal conductivity or heat capacity delayed the rise of the embedded-rebar temperature because the matrix acted as a stronger heat sink, whereas reducing these values accelerated the rebar temperature rise. Variations of the Fe–SMA heat capacity had a direct but smaller influence on the early heating rate, while variations of Fe–SMA thermal conductivity mainly affected the local temperature smoothing inside the rebar domain. This confirms that the calibrated model is sensitive to thermal-property assumptions and should be recalibrated when applied to a different concrete composition, bar geometry or moisture condition.

### 4.3. Input Parameters and User Interaction

The GUI was designed so that all main parameters can be entered directly by the user without modifying the source code. The inputs are grouped into four categories:

- Geometry: beam width and height, rebar diameter, side cover, bottom cover, centre-to-centre spacing of the rebars, and active heated length;
- Electrical loading: current, voltage, heating duration, total simulation time, ambient temperature, calibration target temperature, and calibration target time;
- Material properties: thermal conductivity, density, and specific heat for both UHPFRC and Fe-SMA;
- Numerical and visualisation settings: number of grid points in the *x* and *y* directions, time step, frame-storage interval, convection coefficient, legend temperature limits, and optional automatic pause time.

The default material values used in the present study were selected from available literature. For the UHPFRC matrix, a thermal conductivity of 2.515W/(mK) was adopted from published UHPFRC thermal-property measurements [46], a specific heat of 1009J/(kgK) at room temperature was adopted from UHPC thermal-parameter data [47], and a density of $2500\;\mathrm{kg}/m^{3}$ was used as a representative value within the typical UHPC range of 2400–$2500\;\mathrm{kg}/m^{3}$ [38]. For the Fe-SMA reinforcement, values corresponding to the temperature range close to activation were adopted from the thermophysical measurements of Fe-15Mn-10Cr-8Ni-4Si alloy reported by Rahman [48], namely $\rho =7447.22\;\mathrm{kg}/m^{3}$, $c_{p}=538.54\;J/\left(\mathrm{kg}\;K\right)$, and k=15.62W/(mK) at approximately 199.8 °C. It should be noted that the literature reports some variability in Fe-SMA and concrete thermal parameters depending on alloy composition and concrete type; however, the selected values are appropriate for a calibrated engineering simulation focused on the present activation range.

### 4.4. Program Outputs

After model generation and simulation, the program produces a transient heat-map animation of the beam cross-section, including the outlines of both rebars and a colour legend showing the temperature range. Optional isotherm contours can be superimposed on the temperature field. In parallel, the program generates time–temperature histories for the mean temperature of the embedded Fe-SMA rebars and the maximum concrete temperature. The interface also allows manual time selection, pause/resume functionality, automatic pause at a predefined time instant, export of high-resolution image snapshots, export of GIF animation, and export of the recorded temperature histories for further post-processing. In this way, the tool serves not only as a computational model, but also as a visualization and interpretation environment for the transient activation process.

### 4.5. Illustrative Calibrated Simulation for the Reference Test

To reproduce the reference activation case, the following default inputs were used: I=430Amper, V=2.6Volt, heating time $t_{\mathrm{heat}}=210\;s$, total simulation time $t_{\mathrm{tot}}=420\;s$, and ambient temperature $T_{\infty }=20\;{}^{\circ}C$. These values correspond to a total electrical power of

(9) P=IV=430·2.6=1118W.

Assuming equal division between the two rebars, each bar receives 559W. For a nominal bar diameter of 10mm and an active length of 1.0m, the nominal volumetric power density becomes

(10) $$q_{\mathrm{nom}}^{\prime \prime \prime }=\frac{559}{\pi {\left(0.01\right)}^{2}/4\cdot 1.0}\approx 7.12\times 10^{6}\;W/m^{3}.$$

A unit-gain simulation under these conditions predicted a mean rebar temperature of approximately 70.15 °C at t=210s. Therefore, to match the experimentally observed value of 200 °C at the same time, the source-gain factor was obtained as

(11) g≈3.59,

which yields an effective volumetric heat source of

(12) $$q_{\mathrm{eff}}^{\prime \prime \prime }\approx 2.55\times 10^{7}\;W/m^{3}.$$

The calibrated simulation reproduced the target mean rebar temperature of 200 °C at t=210s. Owing to the geometric symmetry of the model and the equal electrical loading applied to both rebars, the thermal response of the two bars remained nearly identical throughout the heating stage. At the same time, the computed temperature field showed progressive diffusion of heat from the rebars into the surrounding UHPFRC, producing two nearly symmetric thermal zones in the lower part of the beam cross-section. In the calibrated reference case, the mean rebar temperature increased from 20 °C to approximately 128.8 °C after 60s, to 180.1 °C after 150s, and reached 200 °C after 210s. At the same instant, the maximum local concrete temperature adjacent to the rebars reached approximately 188.0 °C, whereas the average temperature of the concrete domain remained much lower, at approximately 31.9 °C. This confirms that the thermal action is highly localized near the activated Fe-SMA rebars, while a large part of the cross-section remains at comparatively low temperature.

After the current cutoff at t=210s, the model predicted a cooling phase controlled only by heat conduction and boundary convection. The mean rebar temperature decreased to approximately 132.6 °C at 240s, 91.0 °C at 300s, and 63.4 °C at the end of the simulated period (420s). At the same instant, the maximum local concrete temperature was approximately 63.4 °C, while the average concrete temperature was approximately 33.3 °C. This indicates that although the thermal peak rapidly decays once the power supply is cut off, residual heat remains concentrated around the bars and continues diffusing into the surrounding matrix for a certain period.

**Figure 11:**
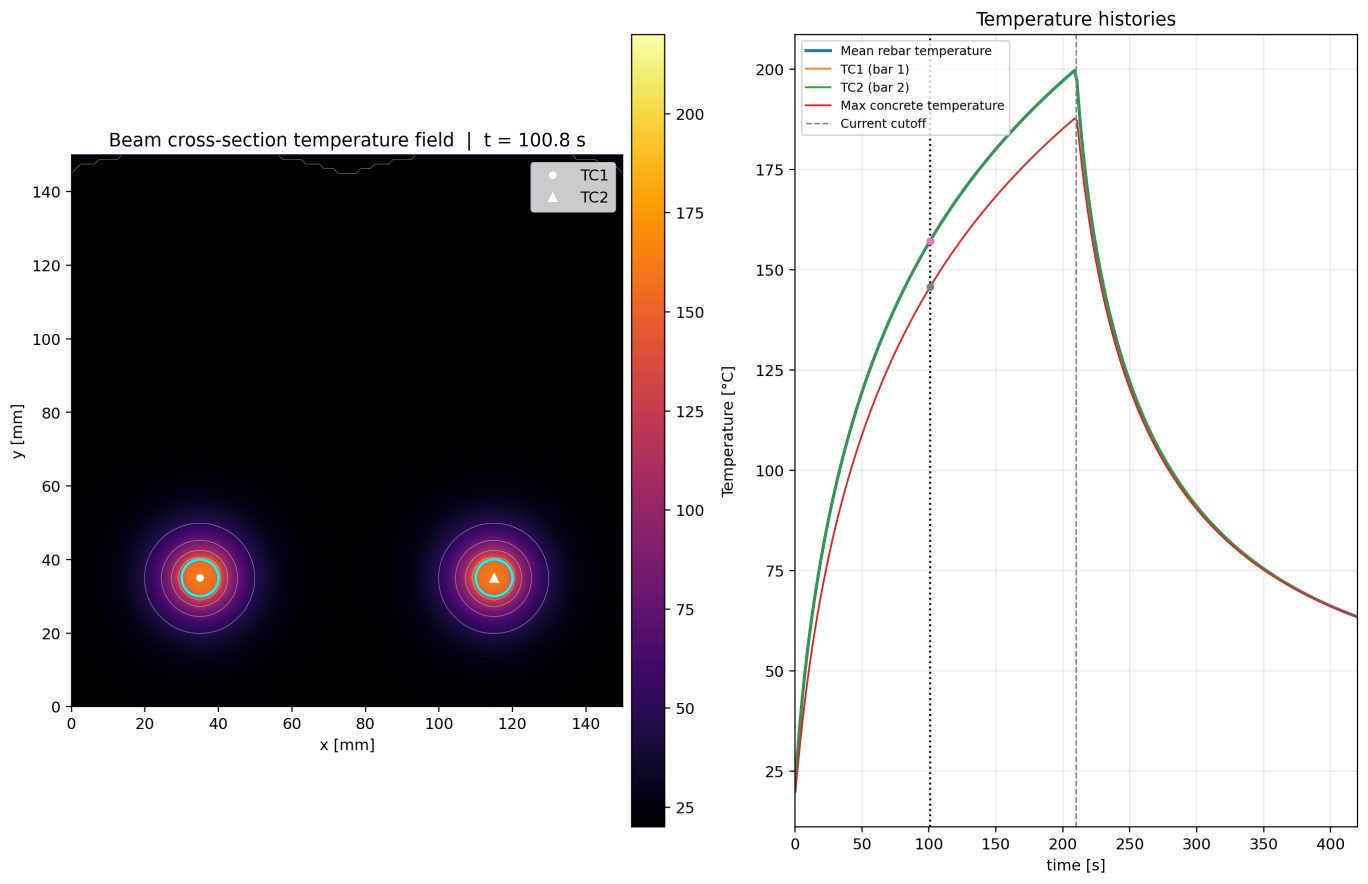
Example output generated by the custom Python electro-thermal simulation tool for the calibrated reference case at t=100.8s. (**Left**): temperature distribution in the beam cross-section. (**Right**): time–temperature histories of the mean Fe-SMA rebar temperature and the maximum local concrete temperature.

**Figure 12:**
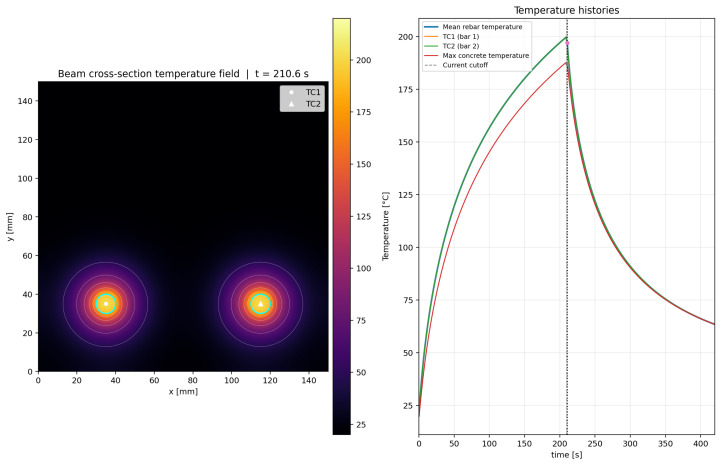
Example output generated by the custom Python electro-thermal simulation tool for the calibrated reference case at t=210.6s, corresponding approximately to the end of the heating phase. (**Left**): temperature distribution in the beam cross-section. (**Right**): time–temperature histories of the mean Fe-SMA rebar temperature and the maximum local concrete temperature; the dashed vertical line indicates the current cutoff.

**Figure 13:**
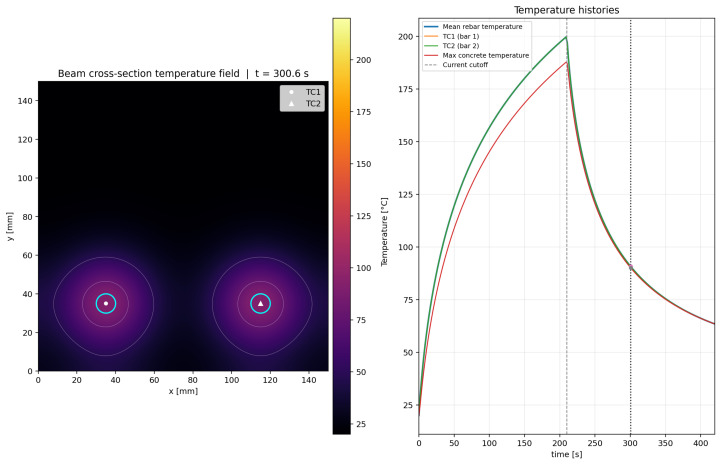
Example output generated by the custom Python electro-thermal simulation tool for the calibrated reference case at t=300.6s during the cooling stage. (**Left**): temperature distribution in the beam cross-section. (**Right**): time–temperature histories of the mean Fe-SMA rebar temperature and the maximum local concrete temperature.

**Figure 14:**
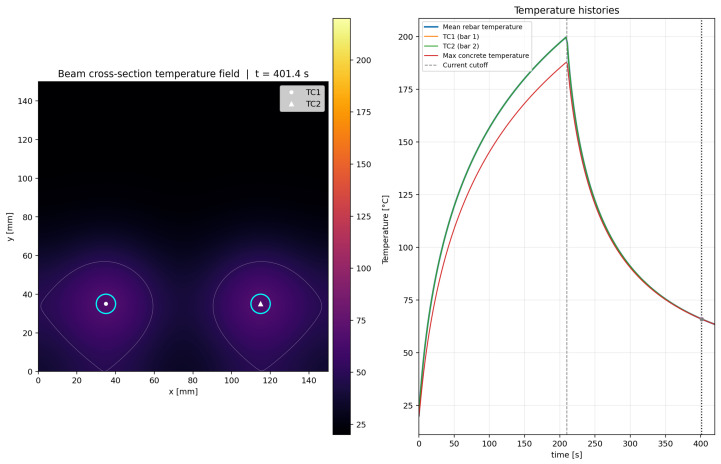
Example output generated by the custom Python electro-thermal simulation tool for the calibrated reference case at t=401.4s in the late cooling stage. (**Left**): temperature distribution in the beam cross-section. (**Right**): time–temperature histories of the mean Fe-SMA rebar temperature and the maximum local concrete temperature.

The simulated evolution of the temperature field is illustrated in Figure 11, Figure 12, Figure 13 and Figure 14 for four representative instants, namely t≈100.8s, 210.6s, 300.6s, and 401.4s. At t≈100.8s (Figure 11), the heating process is still in its rising phase, and the thermal field is strongly concentrated around the two rebars, with steep gradients in the surrounding UHPFRC. At t≈210.6s (Figure 12), i.e., close to the current cutoff, the rebar temperature reaches the target activation level and the surrounding concrete exhibits the largest spatial extent of elevated temperature. At t≈300.6s (Figure 13), after termination of the electrical input, the model predicts a rapid decrease in rebar temperature accompanied by continued redistribution of heat into the surrounding matrix. Finally, at t≈401.4s (Figure 14), the section is already in the late cooling stage, yet the thermal field still shows two distinct residual warm zones around the rebars. Together, these figures demonstrate the three main features of the process: rapid Joule heating of the Fe-SMA rebars, transient heat transfer from the rebars into the UHPFRC matrix, and gradual post-activation cooling controlled by conduction and boundary heat losses.

The proposed Python-based simulation serves as a valuable companion to the experimental study. It allows for rapid parametric assessment of variations in electrical input and thermal properties, provides publication-ready visualizations of heat transfer from Fe-SMA to UHPFRC, and creates an intuitive connection between measured thermocouple data and the transient temperature field within the beam cross-section. It is important to note that since the model was calibrated based on input data, there are slight discrepancies, such as the 10 A difference between the experimental current and the current used in the simulation, as well as a 3 s difference in the time required to reach 200 °C.

The data related to quantitative validation provided in Table 5, deliberately separates calibration accuracy from predictive capability. The small error at the activation target demonstrates that the model reproduces the selected reference case, whereas the sensitivity rows show why independent validation with different current levels, cover depths, rebar diameters and moisture states is still required before the model can be used as a design-predictive tool.

## 5. Discussion and Final Remarks

The present study addresses a practically important stage of Fe-SMA application that is often recognized as critical but is still insufficiently documented in detail, namely the electrical resistance activation of prestrained Fe-SMA rebars after their placement inside a concrete member. In externally accessible systems, the temperature of the active element can usually be observed or controlled more directly. In contrast, for embedded Fe-SMA rebars, the activation process is governed not only by the electrical input itself, but also by the thermal interaction with the surrounding matrix, concrete cover, moisture state, crack pattern, and local boundary conditions. For this reason, the present investigation is important not only as an experimental study on one specific beam configuration, but also as a methodological contribution toward more reliable activation procedures for embedded Fe-SMA systems.

A key contribution of this work lies in the combined interpretation of the experiment and simulation. The experimental results demonstrated that the exposed rebar segment and the embedded working segment cannot be assumed to have the same thermal response. The simulation, although intentionally simplified, supports this interpretation by showing how heat remains strongly localized near the rebars during the heating phase and only gradually diffuses into the surrounding UHPFRC cross-section. In this sense, the numerical model is useful because it provides a spatial interpretation of a process that is measured experimentally only at discrete thermocouple locations. The agreement between the calibrated simulation and the experimental activation target confirms that the model is able to reproduce the main activation milestone and the general evolution of the thermal field in the cross-section.

At the same time, the comparison between test and simulation should be interpreted carefully. The present model is a calibrated two-dimensional engineering tool rather than a fully predictive coupled thermo-electro-mechanical representation. In particular, the source-gain factor is adjusted so that the simulated embedded-rebar temperature reaches the experimentally observed target temperature at the reference activation time. Consequently, the good agreement at that point is partly built into the calibration procedure. The real value of the model therefore lies not in claiming full prediction of all coupled physical processes but in its ability to reproduce the dominant electro-thermal trends, visualize the evolving temperature field, and provide a rational framework for parametric studies. A stronger validation in future work should therefore be based on independent test cases with different current levels, cover depths, bar diameters, or initial moisture conditions, so that calibration and validation are clearly separated.

Another important aspect concerns the thermal condition of the surrounding UHPFRC during activation. The observed moisture release through microcracks indicates that the thermal response of the beam cannot be reduced to simple conductive heating alone. Part of the supplied energy is necessarily consumed by heating the matrix and by moisture-related processes in the pore system. This point is consistent with the broader literature on thermally loaded concrete and UHPC/UHPFRC, where moisture transport, thermal gradients, and pressure build-up are known to influence cracking, damage accumulation, and in more severe cases, even spalling. In the context of the present study, the measured target temperature of approximately 200 °C remained within a moderate activation range compared with fire-type exposure, yet the combination of localized heating, pre-existing cracking, and internal moisture still indicates that the surrounding matrix actively participates in the activation process.

From an engineering point of view, this has two major implications. First, activation procedures should be designed to achieve the temperature required for recovery stress generation in the embedded Fe-SMA, but without unnecessary overheating of exposed bar segments or prolonged heating of the concrete member. Second, the thermal history of the host material should be considered as part of the design problem, especially when activation is performed in dense UHPFRC or in members that have already been pre-cracked. If the heating duration becomes longer than necessary, or if the target temperature is increased substantially above the required activation level, the resulting thermal gradients and moisture effects may intensify microcracking in the cover zone, alter the local bond conditions around the rebars, and reduce the reliability of the activation process itself. In severe cases and under more aggressive thermal exposure, the same mechanisms may increase the risk of local damage or spalling in dense cementitious materials.

Within this context, the benefit of the present simulator becomes particularly clear. Even though it does not yet include all coupled physical mechanisms, it already provides a fast and transparent way to estimate the activation window for a given beam geometry and material set. Instead of relying only on repeated trial-and-error heating tests, the user can vary current, voltage, heating duration, time step, and thermal properties and immediately observe how the rebar temperature and surrounding concrete field evolve. Such a tool is valuable for preselecting experimental parameters, reducing unnecessary overheating, identifying suitable cutoff times, and communicating the activation process in a physically interpretable way. This is especially relevant for future research programs in which several geometric and material variables must be explored efficiently.

The present study also highlights directions for further model development. The most important next step is to move beyond the current calibrated two-dimensional thermal formulation toward a more comprehensive coupled thermo-electro-mechanical description. In particular, future models should consider the longitudinal temperature gradient along the protruding and embedded bar segments, temperature-dependent electrical resistivity of Fe-SMA, heat losses at the electrical terminals, moisture transport in the concrete, and eventually the coupling between thermal activation and mechanical stress transfer. Such extensions would improve the predictive capability of the model and would allow direct estimation not only of temperature evolution, but also of recovery stress development and its transfer to the surrounding UHPFRC.

Overall, the study demonstrates that the activation of embedded Fe-SMA rebars cannot be treated as a purely electrical problem. It is a coupled electro-thermal process influenced by the geometry of the structural element, the thermal inertia and moisture state of the matrix, and the condition of the member at the moment of activation. The experimental program provides the necessary physical evidence for this conclusion, while the custom simulator offers a practical interpretive and parametric tool for extending that evidence into a broader engineering context. For this reason, the combination of test and simulation presented in this work forms a useful basis for the development of safer, more controllable, and more efficient activation procedures for Fe-SMA-reinforced cement-based members.

To place the present activation protocol in the context of previous Fe–SMA activation studies, Table 6 summarizes selected published protocols involving embedded or concrete-coupled Fe–SMA elements. The comparison should be interpreted with caution because the available studies differ substantially in Fe–SMA geometry, cross-sectional area, concrete cover, measurement location, boundary conditions, and activation objective. Nevertheless, a clear tendency can be identified. Small Fe–SMA strips or bars embedded in relatively small concrete specimens have commonly been activated rapidly, often within several seconds to approximately two minutes, using relatively high current densities or concentrated electrical input. In contrast, the present UHPFRC beams had a much larger cross-section, a concrete cover of 30 mm, two embedded 10 mm Fe–SMA rebars, and a pre-cracked, moisture-containing cementitious matrix. Under these conditions, the exposed rebar segment reached the nominal activation temperature much earlier than the embedded region, while the internal thermocouple reached 200 °C only after approximately 213 s. This confirms that activation time cannot be transferred directly from small-scale strip or bar tests to embedded Fe–SMA rebars in UHPFRC members, and that internal temperature monitoring or a validated electro-thermal prediction is required for reliable activation control.

The comparison in Table 6 also explains why the present activation duration is longer than several previously reported values. In earlier strip-based or small-specimen studies, the Fe–SMA element had a smaller thermal mass, shorter heat-flow paths, and a more favorable surface-to-volume ratio, which enabled faster heating. In the present beams, the heat generated in the Fe–SMA rebars was redistributed into a substantially larger UHPFRC volume, while the concrete cover and internal moisture increased the apparent thermal inertia of the system. Therefore, the longer time required to reach 200 °C inside the beam should not be interpreted as inefficient activation alone, but rather as evidence that the embedded thermal state is governed by coupled electrical input, rebar geometry, cover thickness, boundary losses, and matrix condition. This observation is consistent with recent activation-guideline and multiphysical simulation studies, which identify current density, Fe–SMA geometry, and concrete cover as key parameters controlling the temperature and stress fields during resistive activation [27,30].

### Limitations of the Present Study

Several limitations must be considered when interpreting the results. First, the experimental program used one beam size, one concrete cover, one Fe–SMA rebar diameter, one prestrain level and one target activation temperature. Second, fresh-state properties, air content, moisture content, companion mechanical properties, fiber orientation and detailed crack maps were not quantified. Third, recovery stress was inferred from supplier documentation and literature rather than measured directly in the activated beams. Fourth, the electro-thermal model is a calibrated two-dimensional cross-sectional tool; it does not explicitly include 3D heat flow, moisture transport, phase change, temperature-dependent resistivity, terminal losses, contact resistance or mechanical stress transfer. Finally, the numerical model was calibrated to the reference activation test and still requires independent validation under different current levels, cover thicknesses, bar diameters and moisture conditions.

## 6. Conclusions

This study investigated the electrical resistance activation of 4% prestrained Fe–SMA rebars embedded in pre-cracked UHPFRC beams reinforced with two different hooked steel-fiber geometries. Controlled pre-cracking was introduced to reproduce a lightly damaged state before activation, whereas post-activation mechanical reloading was outside the scope of the present work. Within the limits of the experimental and numerical program, the following conclusions can be drawn:

1. The initial flexural response before Fe–SMA activation was affected by the steel-fiber type. Beams reinforced with Dramix 4D fibers reached an average pre-cracking load 9.47% higher than beams reinforced with Dramix 3D fibers. This difference is consistent with the more developed end anchorage of 4D fibers, but it should be interpreted descriptively because crack maps, fiber-orientation data and replicate-level statistical indicators were not available.
2. Electrical resistance heating activated the embedded Fe–SMA rebars, but the exposed and embedded thermal responses were substantially different. Under an average current of approximately 420 A and an average voltage of 2.6 V, the exposed segment reached 200 °C after approximately 77 s, while the embedded control point reached the same target after approximately 213 s. The corresponding thermal lag was approximately 136 s.
3. When the embedded rebar reached the target temperature of 200 °C, the exposed segment had already reached approximately 350 °C. This demonstrates that exposed-bar temperature is not a reliable indicator of the activation state of the working rebar length inside UHPFRC. Internal temperature monitoring or a geometry-specific calibrated thermal model is therefore required for reliable activation control.
4. The measured heating delay reflects the thermal inertia of the UHPFRC cover and the surrounding matrix. Moisture release through microcracks indicates that moisture migration may also contribute to the local thermal response; however, moisture content was not measured directly and this mechanism should therefore be treated as a qualitative interpretation rather than a quantified material parameter.
5. The calibrated Python simulator reproduced the embedded activation target with an activation-time error of approximately 3 s, corresponding to about 1–1.5% of the measured activation time. The source-gain factor should be regarded as an effective calibration parameter, not as a physical heat-loss constant. Sensitivity checks showed that UHPFRC thermal conductivity and heat capacity strongly influence the predicted heating time.
6. From an engineering perspective, reaching the embedded rebar activation temperature is expected to generate recovery stress, promote partial crack closure, improve decompression resistance and enhance serviceability under reloading. These effects were not measured directly in this study and must be verified in future post-activation mechanical tests.
7. The reported activation time is most applicable to members with similar geometry, concrete cover, 10 mm Fe–SMA rebars, similar UHPFRC thermal properties and a target temperature of 200 °C. Larger structural members, bridge-scale systems, longer embedded lengths, larger rebar diameters or thicker covers will require revised calibration and internal temperature control.

## Figures and Tables

**Table 1:** Mix design of the UHPFRC matrix.

Component	Content [kg/m^3^]
CEM II 42.5R cement	700.0
Limestone powder	300.0
Amorphous silica	100.0
Fine quartz sand (0–0.5 mm)	300.0
Coarse quartz sand (0.5–1.0 mm)	710.0
Superplasticizer	16.5
Shrinkage-reducing admixture	16.5
Distilled water	190.0

**Table 2:** Available Fe–SMA rebar characteristics used in the present study (according to supplier documentation).

Parameter	Value or Description
Alloy family	Fe-17Mn-5Si-10Cr-4Ni-1(V, C) (mass%)
Nominal rebar diameter	10 mm
Young’s modulus	70 GPa
Tensile strength	520 MPa
Elongation at fracture	20%
Applied prestrain before casting	4%
Target activation temperature	200 °C
Nominal prestressing force at 200 °C	Approximately 31.5 kN per rebar

**Table 3:** Experimental matrix and specimen designation.

Series	Steel-Fiber Type	Number of Beams
3D	Dramix 3D	6
4D	Dramix 4D	6

**Table 4:** Summary of exposed and embedded Fe–SMA temperature indicators during electrical activation.

Measurement Location	Time to 200 °C [s]	Temperature at Cutoff [°C]	Thermal Lag Relative to TC-1 [s]
TC-1: exposed segment	≈77	≈350	0
TC-2: embedded, control point	≈213	200	≈136
TC-3: embedded, 10 cm by TC-2	≈220	≈195–200	≈140

**Table 5:** Quantitative validation and sensitivity indicators for the calibrated electro-thermal model.

Indicator	Experimental/Baseline Value	Simulation/Sensitivity Result
Embedded activation target	200 °C at ≈213 s	200 °C at ≈210.6 s
Activation-time error	–	≈2.4–3.0 s (≈1–1.5%)
Nominal electrical input	420–430 A, 2.6 V	1.09–1.12 kW
Effective circuit resistance	≈6.2 mΩ	Used only as circuit-level interpretation
UHPFRC *k* decreased/increased by 20%	Baseline ≈ 210 s	≈143 s/≈312 s
UHPFRC $c_{p}$ decreased/increased by 20%	Baseline ≈ 210 s	≈178 s/≈242 s
Fe–SMA *k* decreased/increased by 20%	Baseline ≈ 210 s	≈205 s/≈213 s
Fe–SMA $c_{p}$ decreased/increased by 20%	Baseline ≈ 210 s	≈200 s/≈220 s
Model dimensionality	3D physical beam	2D calibrated cross-section

**Table 6:** Comparison of selected Fe–SMA electrical activation protocols reported in the literature and in the present study.

Study	Test Configuration	Electrical Input	Target Temperature	Heating Time	Relevance to the Current Study
Czaderski et al. & Saeedi et al. [16,30]	Ribbed Fe–SMA strip embedded in a concrete bar; concrete specimen approximately 700 mm × 50 mm × 35 mm; strip cross-section approximately 1.6 mm × 14–16 mm	Approximately 14 A/mm^2^	Power supply stopped at approximately 150 °C; temperature continued to about 160 °C	Approximately 7 s to reach about 160 °C	Demonstrates rapid activation of a thin embedded Fe–SMA strip in a small concrete member; useful as a lower-scale benchmark but not directly transferable to larger UHPFRC beams.
Hong et al. [49]	Near-surface-mounted Fe–SMA strips used for flexural strengthening of RC beams; different strip cross-sectional areas were investigated	Approximately 10 A/mm^2^	160 °C	Approximately 45, 75, and 122 s, depending on Fe–SMA strip cross-section	Shows that even for the same target temperature, activation time depends strongly on Fe–SMA cross-section and thermal environment.
Hong et al. [24]	Fe–SMA rebars embedded as tensile reinforcement in RC beams; square Fe–SMA rebars with different reinforcement areas	Approximately 5 A/mm^2^ during beam activation	Central rebar temperature of 160 °C was associated with a much higher measured temperature at the exposed/threaded end in preliminary calibration	Not reported as a single activation time in the summarized protocol	Important evidence that exposed/end temperatures may differ substantially from the effective internal activation temperature of embedded Fe–SMA rebars.
Zheng et al. [27]	Multiphysical finite-element activation guideline for Fe–SMA embedded in concrete; current density, Fe–SMA geometry, size, and cover depth were parametrically studied	Parametric current-density range, rather than one fixed experimental value	Activation temperature predicted as a function of current density, geometry, and cover depth	Activation time predicted parametrically	Directly supports the need for activation guidelines and confirms that current density, Fe–SMA size/shape, and concrete cover govern the internal temperature field.
Present study	Two 10 mm Fe–SMA rebars embedded in pre-cracked UHPFRC beams with 30 mm concrete cover; beam size 150 mm × 150 mm × 600 mm	Average secondary current of approximately 420 A and voltage of approximately 2.6 V; nominal current density depends on whether the current is normalized by one or both rebars ^a^	200 °C at the internal thermocouple; exposed segment reached 200 °C much earlier	Approximately 77 s at the exposed segment and approximately 213 s at the embedded thermocouple	Shows a pronounced thermal delay between exposed and embedded rebar segments in pre-cracked UHPFRC, demonstrating that exposed-bar temperature is not sufficient for activation control.

^a^ For comparison purposes, normalizing the total current of 420 A by the total nominal area of two 10 mm diameter rebars gives approximately 2.67 A/mm^2^, whereas normalization by one 10 mm rebar gives approximately 5.35 A/mm^2^. Therefore, the directly measured current and voltage are reported, and the current density is treated only as a nominal indicator rather than a strict material parameter.

## Data Availability

The original contributions presented in this study are included in the article. Further inquiries can be directed to the corresponding author.
